# Surgical and health outcomes of non‐ambulatory children with cerebral palsy and severe scoliosis: A population‐based, longitudinal study

**DOI:** 10.1111/dmcn.16473

**Published:** 2025-08-20

**Authors:** Svend Vinje, Terje Terjesen, Joachim Horn, Sandra Julsen Hollung, Thomas Kibsgård

**Affiliations:** ^1^ Division of Orthopaedic Surgery Oslo University Hospital Oslo Norway; ^2^ Institute of Clinical Medicine University of Oslo Oslo Norway; ^3^ Norwegian Quality and Surveillance Registry for Cerebral Palsy, Vestfold Hospital Trust Tønsberg Norway; ^4^ Department of Clinical and Molecular Medicine Norwegian University of Science and Technology Trondheim Norway

## Abstract

**Aim:**

To evaluate medium‐term surgical outcomes, complications, mortality, and health‐related quality of life (HRQoL) in non‐ambulatory children with cerebral palsy (CP) and severe scoliosis, and to analyse outcomes and mortality rates in children who had not undergone surgery.

**Method:**

Data on non‐ambulatory children with CP and severe scoliosis born from 2002 to 2008 were extracted from the Norwegian Quality and Surveillance Registry for Cerebral Palsy. Seventy‐five children (44 males, 31 females) were included. Thirty‐eight (51%; mean age at surgery 14 years 4 months; SD = 2 years 4 months; range = 8–18 years) underwent surgical correction and posterior spinal fusion, with a mean preoperative Cobb angle of 90° (range = 49°–140°), while 37 (49%) children had non‐surgical treatment. HRQoL was measured with the Caregiver Priorities and Child Health Index of Life with Disabilities (CPCHILD).

**Results:**

Eighteen children (47%) had postoperative complications; 5 of 38 (13%) children underwent further surgery. Surgical treatment improved sitting posture and back pain. The mean CPCHILD score was 49.0 (range = 19–84). Among non‐surgically treated children, 15 of the 31 children considered too fragile to undergo spinal surgery (48%) died during the follow‐up; the mean CPCHILD score for the remaining children was 36.4 points (range = 9–59).

**Interpretation:**

Although surgical correction of scoliosis in non‐ambulatory children with CP carried a high risk of complications and re‐operations, it resulted in improved sitting posture and reduced back pain. Children who were not eligible for surgical treatment had a high mortality rate.

## Abstract 2

Plain language summary: https://onlinelibrary.wiley.com/doi/10.1111/dmcn.70010

**Abbreviations**
CPCHILD: Caregiver Priorities and Child Health Index of Life with Disabilities
HRQoL: health‐related quality of life
ICU: intensive care unit
NorCP: Norwegian Quality and Surveillance Registry for Cerebral Palsy

**What this paper adds**

- Non‐ambulatory children with cerebral palsy are at a high risk of developing severe scoliosis.
- Children who underwent surgery for severe scoliosis had a high risk of postoperative complications and re‐operations.
- Children who underwent spinal fusion had improvements in sitting posture and reduced back pain.
- Children not eligible for surgical treatment had a high mortality rate.

Scoliosis is common and develops early and progressively in non‐ambulatory children with cerebral palsy (CP).^1^, ^2^, ^3^ Children classified in Gross Motor Function Classification System^4^ (GMFCS) levels IV and V often develop severe scoliosis, which is associated with decreased respiratory function and pelvic obliquity with coronal or sagittal imbalance (or both), leading to sitting difficulties.^5^ Many of these children require surgical correction through posterior spinal fusion when their scoliosis exceeds 50°, ideally not later than 70° to 90° for children classified in GMFCS levels IV and V.^6^ Scoliosis can occur together with hip dysplasia or pelvic obliquity (or both), which may further compromise the child's functional capabilities.^7^, ^8^

Spinal deformity correction for children with CP is associated with high complication rates and challenging ethical considerations.^9^ Despite this, caregivers are usually satisfied, and these children's health‐related quality of life (HRQoL) improves after spinal fusion in the short term.^10^, ^11^ Although improvement in HRQoL 2 years after surgery in non‐ambulatory children has been reported,^11^, ^12^ studies of long‐term outcomes are lacking. In the longest follow‐up study of HRQoL after spinal fusion, there was an increase in HRQoL scores 1 year after surgery, but the scores essentially returned to baseline after 5 years.^13^ To explore the outcomes of spinal deformity correction over a longer time on children's general health, functional abilities, and well‐being, a longer follow‐up is needed.

This study evaluated medium‐term outcomes in non‐ambulatory children with CP and severe scoliosis who were treated with surgical correction of their scoliosis, including postoperative complications, mortality, and HRQoL, as well as children who were not treated surgically regarding mortality and HRQoL.

## METHOD

### Study design

Non‐ambulatory children with CP born between 1st January 2002 and 31st December 2008 were consecutively identified using the Norwegian Quality and Surveillance Registry for Cerebral Palsy (NorCP) as of 31st December 2020. NorCP was established in 2001 and is a national medical quality registry and follow‐up programme for children with CP. Medical data are recorded at three time points: at diagnosis; at the age of 5 years (when the diagnosis is confirmed); and at 15 to 17 years. From 2006, NorCP has included longitudinal registration. This includes physiotherapy measurements and interventions, and orthopaedic surgeries.^14^ In this study, NorCP was linked to the Norwegian Cause of Death Registry to extract information on the causes of death.

In total, 157 non‐ambulatory children with CP were identified. Inclusion criteria included children classified in GMFCS levels IV and V with severe scoliosis (Cobb angle ≥ 40°). Seventy‐five children filled these criteria (44 males [59%] and 31 [41%] females). Most had bilateral spastic CP, were classified in GMFCS level V, and had a gastrostomy tube and epilepsy. Children either underwent surgical correction and spinal fusion at Oslo University Hospital between 2014 and 2023 (*n* = 38) or did not receive surgical treatment (*n* = 37). Table 1 describes the baseline demographic and clinical features for both sets of children. A flow chart of the children is shown in Figure S1.

**TABLE 1:** Clinical features of 75 non‐ambulatory children with cerebral palsy (CP) and severe scoliosis (Cobb angle ≥ 40°).

Characteristic	Surgical treatment	Non‐surgical treatment
Number of children	38 (51)	37 (49)
Sex		
Male	18 (47)	26 (70)
Female	20 (53)	11 (30)
CP subtype		
Bilateral spastic	27 (71)	33 (89)
Dyskinetic	11 (29)	3 (8)
Ataxic	0 (0)	1 (3)
GMFCS level		
IV	6 (16)	2 (5)
V	32 (84)	35 (95)
Epilepsy		
Yes	28 (74)	29 (78)
No	5 (13)	2 (5)
Unknown	5 (13)	6 (16)
Gastrostomy tube		
Yes	26 (68)	29 (78)
No	12 (32)	8 (22)
Nissen fundoplication		
Yes	4 (11)	4 (11)
No	34 (89)	33 (89)
Age at surgery, years:months (SD) (range)	14:4 (2:4) (8–18)	–
Age at CPCHILD, years:months (SD) (range)	19:8 (1:7) (15–22)	19:7 (1:6) (17–22)
Preoperative Cobb angle, mean (SD) (range)	90° (19.5) (49°–140°)	–
Cobb angle at the last follow‐up, mean (SD) (range)	42° (16.3) (17°–95°)	90° (22.1) (56°–137°)

*Note*: Data are *n* (%) unless otherwise stated. Abbreviations: CP, cerebral palsy; CPCHILD, Caregiver Priorities and Child Health Index of Life with Disabilities; GMFCS, Gross Motor Function Classification System.

### Variables

Information on demographics was extracted from NorCP. Clinical data, also extracted from NorCP, included GMFCS level and CP subtype, classified according to the Surveillance of Cerebral Palsy in Europe guidelines as bilateral spastic, dyskinetic, and ataxic CP.^15^ Comorbidities included the use of a gastrostomy tube for feeding, epilepsy, and Nissen fundoplication, defined as either yes or no. Information on the child's seating posture and back pain was extracted from either the NorCP physiotherapy notes or hospital electronic case records. Radiographic measurements and surgical characteristics were extracted from the hospital's electronic case records. Before and after surgery, Cobb angles were measured on X‐rays taken in the sitting position, with support provided by a caregiver if needed. Surgical characteristics included surgical approach, use of computer‐assisted navigation, fused spinal levels, pelvic fixation, surgical time, and estimated blood loss. Intraoperative and postoperative complications consisted of cardiopulmonary, gastrointestinal, and neurological complications, and surgical wound infection. Postoperative parameters were hours spent in the intensive care unit (ICU) and length of hospitalization.

Data on mortality were extracted from the Norwegian Cause of Death Registry and included the cause of death, classified using the International Classification of Diseases, 10th Revision codes.

### Health‐related quality of life

Information on HRQoL was collected from the caregivers of living children at the end of the study (May 2024). They were sent the Caregiver Priorities and Child Health Index of Life with Disabilities (CPCHILD) questionnaire. CPCHILD is internationally validated^16^ and consists of six domains and 37 items: (1) personal care/activities of daily living (nine items); (2) positioning, transferring, mobility (eight items); (3) comfort, emotions, and behaviour (nine items); (4) communication and social interaction (seven items); (5) health (three items); and (6) overall quality of life (one item). Standardized scores were calculated for each of the domains, ranging from 0 (worst) to 100 (best) points. One combined total score per child was calculated. In addition, three additional questions were sent to the caregivers of children who were surgically treated: (1) In general, how satisfied are you with the surgery? (scale from 0 to 10 points); (2) Would you let your child undergo the surgery again, given the opportunity to make this decision again? (yes or no); and (3) Would you recommend the surgery to others in the same situation? (yes or no).

### Statistics

SPSS v29 (IBM Corp., Armonk, NY, USA) was used for the statistical analysis. Mean values, SDs, and ranges were used to describe the demographic and clinical features.

### Ethics

NorCP is a consent‐based national medical quality registry and is governed by the Regulation on Medical Quality Registries. This study was approved by the Regional Committees for Medical and Healthcare Research Ethics (no. 107726), the University of Oslo Privacy and Data Protection Officer, and the institutional review board of the Division of Orthopaedic Surgery at Oslo University Hospital. The NorCP written informed consent form obtained from the children or their caregivers was approved for this study and to publish the results.

## RESULTS

### Surgically treated children

Thirty‐eight of 75 (51%) children underwent spinal fusion. The indications for surgery were increasing scoliosis and seating difficulties in 30 children, increasing scoliosis in six, and planned surgery after some years with a ‘growing rod’ in two children. Preoperatively, 30 of 38 (79%) children had problems with sitting balance, six (16%) had no problems, while two children had missing information. Postoperatively, seating posture improved in 29 of 30 (97%) children. Twenty‐two of 38 (58%) children reported back pain before surgery; 15 of them reported relief from their pain after surgery, while five (13%) still had back pain; information about two children was missing.

**FIGURE 1:**
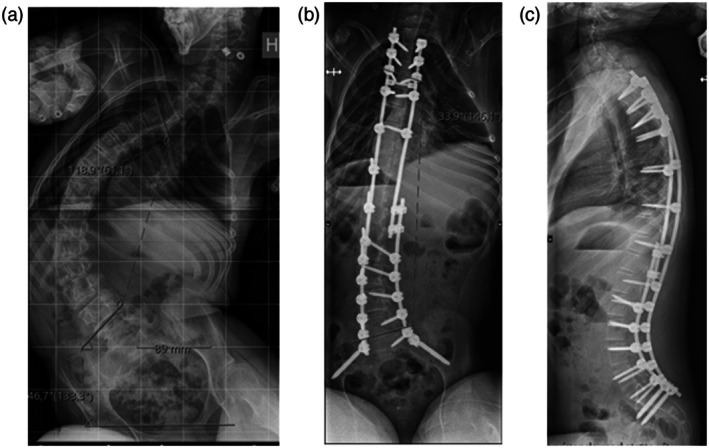
Preoperative posteroanterior X‐ray of a 16‐year‐old female with cerebral palsy and severe scoliosis (Cobb angle = 118°), classified in Gross Motor Function Classification System level V (a). First postoperative posteroanterior (b) and lateral (c) X‐rays 6 months after posterior spinal fusion from the second thoracic (T2) vertebra to the pelvis, according to the spinal implant: MESA 5.5 titanium (Stryker, Michigan, USA). The postoperative Cobb angle was 34°.

The mean preoperative Cobb angle was 90° (SD = 19.5; range = 49°–140°) and the mean age at surgery was 14 years 4 months (SD = 2 years 4 months; range = 8–18 years). At the last radiographic follow‐up, at the mean age of 18 years 8 months (SD = 2 years 6 months; range = 11–22 years), the mean Cobb angle was 42° (SD = 16.3°; range = 17°–95°). All children had posterior segmental instrumentation, with use of pedicle screws, hooks, and sublaminar wires for connection to the main construct (Figure 1). The mean number of levels fused was 15 (SD = 0.9; range = 12–17). Thirty‐five children (92%) had pelvic fixation.

The mean surgical time was 301 minutes (SD = 60.7 minutes; range = 207–423 minutes). Postoperatively, children were in the ICU for a median of 19 hours (range = 2–288 hours); six children had an extended length of stay in the ICU (> 48 hours). The mean length of stay in the hospital was 11 days (SD = 5.0 days; range = 6–29 days).

Eighteen of 38 children (47%) had 20 postoperative complications: 11 (29%) had a respiratory complication; two (5%) suffered a urinary tract infection; and two (5%) had a postoperative epileptic seizure. Five re‐operations (13%) were performed, two because of surgical site infections and three because of implant‐related (mechanical) issues. One child had a two‐stage procedure because of challenging placement of an intraoperative respiratory airway tube at the primary surgery.

Four of 38 children (11%) died at a mean of 928 days (SD = 671.9 days; range = 161–1780 days) postoperatively, at a mean age of 15 years 11 months (SD = 2 years 2 months; range = 14–19 years). The causes of death were pneumonia and other respiratory issues in three children and epilepsy in one child. There were no cases of perioperative deaths or mortality related to the index surgery.

### Non‐surgically treated children

Thirty‐seven children (49%) did not receive surgical treatment despite being evaluated annually by a multidisciplinary team. Six children had stable scoliosis curves below 60° (range = 42°–57°) and did not require surgery. Five children died before the age of 12 years and, because of their overall fragility, were never considered for surgical intervention. Twenty‐six of 37 children had a progressive curve and were evaluated for surgery; however, for several reasons, they were declined. At the last follow‐up, these children had a mean Cobb angle of 90.2° (SD = 22.1°; range = 56°–137°); mean age was 17 years 2 months (SD = 3 years 11 months; range = 11–22 years).

Respiratory conditions were the most common contraindication for surgical treatment (14 of 26 [54%] children). Other comorbidities or overall frail health were present in 10 (38%). In two children, the parents refused surgical treatment recommended by the multidisciplinary team (last Cobb angles 56° and 117°). Of the 31 children considered too fragile to undergo surgery, 15 (48%) died during the follow‐up, at a mean age of 14 years 1 month (SD = 4 years 4 months; range = 5–20 years). The most common cause of death was pneumonia or respiratory diseases (11 of 16 children, 69%). The other five causes were recorded as an instantaneous death, infectious disease, circulatory system disorder, oesophagus or feeding disorder, and unknown.

### Health‐related quality of life

During the follow‐up, 20 children died. Thus, 55 children (34 surgically treated and 21 non‐surgically treated) were eligible for the CPCHILD analysis. Among these, 30 caregivers (88%) of surgically treated children completed the CPCHILD questionnaire and responded to the three additional questions, while 17 caregivers (81%) of non‐surgically treated children completed the questionnaire (Figure 1). CPCHILD scores were not directly compared using *p*‐values as a direct comparison between surgically treated and non‐surgically treated children was not appropriate because of greater medical complexity and frailty in non‐surgically treated children. Most caregivers, regardless of whether children were surgically treated or not, were females (*n* = 36) with a college degree (*n* = 25), who worked full time (*n* = 33).

Children in the surgically treated cohort had a mean age of 19 years 8 months (SD = 1 year 7 months; range = 15–22 years) at the time of the CPCHILD evaluation. The mean postoperative follow‐up was 5 years 1 month (SD = 2 years 4 months; range = 1–10 years). They had a mean overall CPCHILD score of 49.0 points (SD = 15.1 points; range = 19–84 points) (Table 2). Among the specific domain scores, the lowest score was in personal care/activities of daily living (35.1 points) and positioning, transferring, mobility (36.5 points). Most caregivers would choose to have their child undergo surgery again (*n* = 26 of 30 [87%]), as well as recommending the surgery to others in the same situation (*n* = 29 of 30 [97%]).

**TABLE 2:** CPCHILD questionnaire at the last follow‐up for non‐ambulatory children with cerebral palsy and severe scoliosis (Cobb angle ≥ 40°).

Domain number	Description	Surgically treated group (*n* = 30), mean (SD)	Non‐surgically treated group (*n* = 15),^a^ mean (SD)
1	Personal care/activities of daily living	35.1 (22.0)	34.0 (12.8)
2	Positioning, transferring, mobility	36.5 (25.0)	24.6 (15.2)
3	Comfort and emotions	63.8 (30.8)	55.1 (30.3)
4	Communication and social interaction	40.1 (23.8)	26.7 (20.6)
5	Health	60.1 (19.3)	44.0 (17.8)
6	Overall quality of life	58.9 (22.3)	40.0 (26.2)
Total score		49.0 (15.1)	36.4 (13.4)

^a^ Two children with stable scoliosis were not included in the non‐surgically treated group. Abbreviation: CPCHILD, Caregiver Priorities and Child Health Index of Life with Disabilities.

Of the non‐surgically treated participants, 17 CPCHILD evaluations were completed. Among these, 15 had contraindications for spinal surgery and two had stable scoliosis (< 60°) The mean age of the 15 children with contraindications was 19 years 7 months (SD = 1 year 6 months; range = 17–22 years) and the mean Cobb angle was 102.6° (SD = 18.1°; range = 72°–137°) at the final follow‐up. They had a mean CPCHILD total score of 36.4 points (SD = 13.4 points; range = 9–59 points) (Table 2). The lowest scores were in domain 2, positioning, transferring, mobility (24.6 points), and domain 4, communication (26.7 points). The mean total CPCHILD scores in two children with stable scoliosis (< 60°) were 53.1 and 56.1 points.

## DISCUSSION

Non‐ambulatory children with CP have a high risk of developing severe scoliosis. Approximately half of the children in NorCP who were classified in GMFCS levels IV and V developed severe scoliosis with a Cobb angle ≥ 40°.^3^ When progressive scoliosis reaches this magnitude, a decision regarding surgical intervention should be made. In this study, half of the children with severe scoliosis underwent spinal fusion. Most children who were not surgically treated were advised against major surgery because of respiratory disorders or because the overall burden of impairments was considered too high.

The postoperative complication rate is high in children classified in GMFCS levels IV and V undergoing spinal fusion. A systematic review reported an overall complication risk varying from 11% to 71%,^17^ while another review found a mean complication rate of 33% (range = 18%–81%) during the first 3 months after surgery.^9^ Thus, our postoperative complication rate of 47% was in agreement with previous studies. Pulmonary and respiratory problems were the most frequent complications and occurred in 27% of children, which was high but still lower than in most other studies, where respiratory problems ranged from 27% to 57% according to Legg et al.^17^ The wound infection rate was 13%, which is similar to the range of superficial and deep wound infections of 9% to 13% reported previously.^12^, ^18^, ^19^ Three children underwent further surgery because of hardware‐related failure, which is similar to the experience of others.^20^, ^21^, ^22^, ^23^ During the hospital stay, 16% of children had postoperative difficulties that required an extended length of stay in the ICU. Although several children had a prolonged stay in the ICU, all survived surgery and there was no mortality related to the primary surgery or the hospitalization.

In our study, the mortality rate of children surgically treated was 11% at the follow‐up. This is comparable to earlier reports where mortality rates ranged from 3% to 19%.^17^, ^24^, ^25^ The mortality rate (48%) among children who were too fragile for surgical intervention was much higher. Cassidy et al.^26^ studied an institutionalized cohort and found a 39% mortality rate, which is consistent with our study. Most deaths were caused by respiratory diseases (primarily pneumonia), which is in agreement with a recent systematic review of mortality in children with CP.^27^ The mortality rates in our study suggest that surgery would have decreased mortality among non‐surgically treated children. On the other hand, the high mortality may indicate that the initial evaluation was correct and that these children were too impaired to receive a major surgical procedure.

The long‐term functional outcome after spinal fusion in children with CP remains controversial.^12^ Seating posture is a key function for non‐ambulatory children. Previous studies reported an improvement in sitting balance after surgery for scoliosis.^19^, ^25^, ^28^, ^29^ Our results confirmed this positive effect. Thirty of the 38 children in our surgical cohort had problems with seating posture preoperatively; all except one experienced improvement after surgery. Spine‐related pain is an important factor; in some studies, it has been shown to affect HRQoL and participation for children with CP.^19^, ^28^, ^29^ In the present study, 22 children had preoperative back pain, which improved in approximately two‐thirds. This reduction of pain is in accordance with Sewell et al.,^29^ who studied non‐ambulatory children using CPCHILD preoperatively and at the follow‐up 2 years after surgery. They showed an improvement in overall CPCHILD scores, which was mainly due to improvements in sitting balance and back pain.

This study used the CPCHILD questionnaire, which is an accepted condition‐specific instrument measuring the HRQoL of children with CP.^16^ The mean total score among surgically treated children (49.0 points) is within the range of the reference scores of 44 and 56 points at GMFCS levels IV and V respectively, according to the CPCHILD reference manual.^16^ Previous studies found higher total scores ranging from 50 to 59 points 2 years postoperatively.^11^, ^12^, ^28^, ^29^, ^30^ Studies with both preoperative and 2‐year postoperative CPCHILD registrations found an improvement in total scores ranging from 3 to 7 points.^11^, ^12^, ^28^, ^30^, ^31^ Two studies with a 5‐year follow‐up, which is the longest previous follow‐up of CPCHILD, reported somewhat divergent results. Whereas DiFazio et al.^13^ reported an initial improvement in total score, but no significant change from preoperative score to the score at 5 years, Miyanji et al.^31^ found an increase of seven points 1 year postoperatively, with the improvement maintained at 2 and 5 years.

The mean total CPCHILD score among children not eligible for surgical treatment was 36 points, which is significantly lower than that of children treated surgically, and markedly lower than the total scores of 50 and 53 points respectively in the non‐surgically treated groups of Sewell et al.^29^ and Cahill et al.^28^ The reasons for these differences were probably the different study design and selection bias. While our study was population‐based, the other two were based on hospital referrals for scoliosis surgery. Thus, children with CP with many comorbidities and impairments would probably not be referred because their health was considered too frail for spine surgery. Children who underwent surgery demonstrated notably elevated scores in domains 5 (health) and 6 (overall quality of life) of the CPCHILD questionnaire. The mean score in domain 3 (comfort and emotions) of 63.8 points among surgically treated children was markedly lower than the 84.7 points reported by DiFazio et al.,^13^ while the scores of the other domains were comparable. We have no plausible reasons for the difference in domain 3. The overall satisfaction among caregivers of children who were surgically treated was high, based on three additional questions, which is in agreement with other studies.^10^, ^32^

This study has several limitations. First, it included a relatively small number of non‐ambulatory children with CP and severe scoliosis. The high mortality rate further reduced the number of children available for the CPCHILD analyses. Second, the study was retrospective, with no randomization of children to the two cohorts. As the children in the non‐surgically treated cohort were never candidates for surgery, it would be inappropriate to use them as the control group. Third, we were not able to detect changes in CPCHILD scores over time because there was no preoperative analysis; this reduced the possibility of conducting an effectiveness analysis of the surgical intervention. Fourth, the CPCHILD questionnaire has some limitations, such as lack of evaluation of pulmonary function and only one question on seating posture (domain 2, positioning, transferring, mobility). Fifth, we were not able to include pelvic obliquity in the radiographic measurements because we found sitting position pelvic obliquity rather unreliable; the necessary landmarks were not always clearly depicted on the spine X‐rays. Finally, clinical data were based on subjective grading from the NorCP registrations and hospital case records, which led to some missing data. A strength of this study is that it was population‐based; the NorCP includes more than 90% of children with CP in Norway.^33^ Additionally, data were collected nationwide according to international standards and there was a relatively long follow‐up from the time of diagnosis of scoliosis to the last follow‐up. At one specialized hospital, all children were treated surgically, which strengthened data collection postoperatively.

## CONCLUSION

Of 75 non‐ambulatory children with severe scoliosis identified from our national NorCP registry, 38 were surgically treated with curve correction and spinal fusion. They faced substantial risks of postoperative complications and re‐operations. Nevertheless, surgery had a good effect on sitting posture and back pain, with caregivers reporting a high degree of satisfaction with surgery. The most common contraindication among children considered ineligible for surgery were respiratory and pulmonary conditions, as well as reduced general health, which led to a high mortality rate (48%).

## FUNDING INFORMATION

Open access funding provided by Oslo University Hospital (incl University of Oslo).

## CONFLICT OF INTEREST STATEMENT

None.

## Supporting information


**Figure S1:** Inclusion and exclusion criteria for non‐ambulatory children with cerebral palsy and severe scoliosis.

## Data Availability

The data that support the findings of this study are available on request from the corresponding author. The data are not publicly available due to privacy or ethical restrictions.

## References

[1] Willoughby KL , Ang SG , Thomason P , Rutz E , Shore B , Buckland AJ , et al. Epidemiology of scoliosis in cerebral palsy: A population‐based study at skeletal maturity. J Paediatr Child Health. 2022; 58: 295–301. 34453468 10.1111/jpc.15707 PMC9291795

[2] Hägglund G , Pettersson K , Czuba T , Persson‐Bunke M , Rodby‐Bousquet E . Incidence of scoliosis in cerebral palsy. Acta Orthop. 2018; 89: 443–7. 29537343 10.1080/17453674.2018.1450091 PMC6600133

[3] Vinje S , Terjesen T , Kibsgård T . Scoliosis in children with severe cerebral palsy: A population‐based study of 206 children at GMFCS levels III–V. Eur Spine J. 2023; 32: 4030–4036. 37532910 10.1007/s00586-023-07868-1

[4] Palisano RJ , Rosenbaum P , Bartlett D , Livingston MH . Content validity of the expanded and revised Gross Motor Function Classification System. Dev Med Child Neurol. 2008; 50: 744–50. 18834387 10.1111/j.1469-8749.2008.03089.x

[5] Majd ME , Muldowny DS , Holt RT . Natural history of scoliosis in the institutionalized adult cerebral palsy population. Spine. 1997; 22: 1461–6. 9231964 10.1097/00007632-199707010-00007

[6] Hollenbeck SM , Yaszay B , Sponseller PD , Bartley CE , Shah SA , Asghar J , et al. The pros and cons of operating early versus late in the progression of cerebral palsy scoliosis. Spine deform. 2019; 7: 489–93. 31053320 10.1016/j.jspd.2018.09.002

[7] Helenius IJ , Viehweger E , Castelein RM . Cerebral palsy with dislocated hip and scoliosis: what to deal with first? J Child Orthop. 2020; 14: 24–9. 32165978 10.1302/1863-2548.14.190099 PMC7043124

[8] Terjesen T , Vinje S , Kibsgård T . The relationship between hip displacement, scoliosis, and pelvic obliquity in 106 nonambulatory children with cerebral palsy: A longitudinal retrospective population‐based study. Acta Orthop. 2024; 95: 67–72. 38288626 10.2340/17453674.2024.39915 PMC10825870

[9] Whitaker AT , Sharkey M , Diab M . Spinal fusion for scoliosis in patients with globally involved cerebral palsy: An ethical assessment. J Bone Joint Surg. 2015; 97: 782–7. 25948526 10.2106/JBJS.N.00468

[10] Tsirikos AI , Chang W‐N , Dabney KW , Miller F . Comparison of parents' and caregivers' satisfaction after spinal fusion in children with cerebral palsy. J Pediatr Orthop. 2004; 24: 54–8. 14676534 10.1097/00004694-200401000-00010

[11] DiFazio RL , Miller PE , Vessey JA , Snyder BD . Health‐related quality of life and care giver burden following spinal fusion in children with cerebral palsy. Spine. 2017; 42: 733–9. 27792122 10.1097/BRS.0000000000001940

[12] Miller DJ , Flynn JJM , Pasha S , Yaszay B , Parent S , Asghar J , et al. Improving health‐related quality of life for patients with nonambulatory cerebral palsy: who stands to gain from scoliosis surgery? J Pediatr Orthop. 2020; 40: 186–92. 10.1097/BPO.0000000000001424 31306277

[13] DiFazio RL , Vessey JA , Miller PE , Snyder BD , Shore BJ . Health‐related quality of life and caregiver burden after hip reconstruction and spinal fusion in children with spastic cerebral palsy. Dev Med Child Neurol. 2022; 64: 80–7. 34296760 10.1111/dmcn.14994

[14] Hollung SJ , Jahnsen RB , Klevberg GL , Kløve N , Andersen GL . The impact of longitudinal surveillance of individuals with cerebral palsy in Norway; a 20‐year quality registry and follow‐up program perspective. Norsk Epidemiologi. 2023; 31: 15–23.

[15] Shaunak M , Kelly VB . Cerebral palsy in under 25 s: assessment and management (NICE Guideline NG62). Arch Dis Child Educ Pract. 2018; 103: 189–93. 10.1136/archdischild-2017-312970 29056589

[16] Narayanan UG , Fehlings D , Weir S , Knights S , Kiran S , Campbell K . Initial development and validation of the Caregiver Priorities and Child Health Index of Life with Disabilities (CPCHILD). Dev Med Child Neurol. 2006; 48: 804–12. 16978459 10.1017/S0012162206001745

[17] Legg J , Davies E , Raich AL , Dettori JR , Sherry N . Surgical correction of scoliosis in children with spastic quadriplegia: benefits, adverse effects, and patient selection. Evid Based Spine Care J. 2014; 5: 038–51. 10.1055/s-0034-1370898 PMC3969433 24715871

[18] Cloake T , Gardner A . The management of scoliosis in children with cerebral palsy: A review. J Spine Surg. 2016; 2: 299–309. 28097247 10.21037/jss.2016.09.05 PMC5233861

[19] Roberts SB , Tsirikos AI . Factors influencing the evaluation and management of neuromuscular scoliosis: A review of the literature. J Back Musculoskelet. 2016; 29: 613–23. 10.3233/BMR-160675 26966821

[20] Tsirikos AI . Development and treatment of spinal deformity in patients with cerebral palsy. Indian J of Orthop. 2010; 44: 148–58. 20419001 10.4103/0019-5413.62052 PMC2856389

[21] Nectoux E , Giacomelli M , Karger C , Herbaux B , Clavert J . Complications of the Luque–Galveston scoliosis correction technique in paediatric cerebral palsy. Orthop Traumatol Surg Res. 2010; 96: 354–61. 20471343 10.1016/j.otsr.2010.01.004

[22] Vialle R , Delecourt C , Morin C . Surgical treatment of scoliosis with pelvic obliquity in cerebral palsy: the influence of intraoperative traction. Spine. 2006; 31: 1461–6. 16741455 10.1097/01.brs.0000219874.46680.87

[23] Sink EL , Newton PO , Mubarak SJ , Wenger DR . Maintenance of sagittal plane alignment after surgical correction of spinal deformity in patients with cerebral palsy. Spine. 2003; 28: 1396–403. 12838097 10.1097/01.BRS.0000067088.99346.73

[24] Ahonen M , Helenius I , Gissler M , Jeglinsky‐Kankainen I . Mortality and causes of death in children with cerebral palsy with scoliosis treated with and without surgery. Neurology. 2023; 101: 1787–92. 10.1212/WNL.0000000000207796 PMC10634643 37679048

[25] Comstock CP , Leach J , Wenger DR . Scoliosis in total‐body involvement cerebral palsy: analysis of surgical treatment and patient and caregiver satisfaction. Spine. 1998; 23: 1412–24. 9654634 10.1097/00007632-199806150-00022

[26] Cassidy C , Craig CL , Perry A , Karlin LI , Goldberg MJ . A reassessment of spinal stabilization in severe cerebral palsy. J Pediatr Orthop. 1994; 14: 731–9. 7814585 10.1097/01241398-199414060-00008

[27] Aldharman SS , Alhamad FS , Alharbi RM , Almutairi YS , Alhomsi MWM , Alzahrani SA , et al. Risk factors for mortality in patients with cerebral palsy: A systematic review and meta‐analysis. Cureus. 2023; 15: 39327. 10.7759/cureus.39327 PMC10292172 37378195

[28] Cahill PJ , Narayanan U , Bowen M , Sarkar S , Pahys JM , Miyanji F , et al. Impact of spinal deformity and surgery on health‐related quality of life in cerebral palsy: A multicenter prospective controlled trial. J Pediatr Orthop. 2024; 44: 901–7. 10.1097/BPO.0000000000002774 39077879

[29] Sewell MD , Malagelada F , Wallace C , Gibson A , Noordeen H , Tucker S , et al. A preliminary study to assess whether spinal fusion for scoliosis improves carer‐assessed quality of life for children with GMFCS level IV or V cerebral palsy. J Pediatr Orthop. 2016; 36: 299–304. 25851675 10.1097/BPO.0000000000000447

[30] Jain A , Sponseller PD , Shah SA , Samdani A , Cahill PJ , Yaszay B , et al. Subclassification of GMFCS level‐5 cerebral palsy as a predictor of complications and health‐related quality of life after spinal arthrodesis. J Bone Joint Surg 2016; 98: 1821–8. 27807115 10.2106/JBJS.15.01359

[31] Miyanji F , Nasto LA , Sponseller PD , Shah SA , Samdani AF , Lonner B , et al. Assessing the risk–benefit ratio of scoliosis surgery in cerebral palsy: Surgery is worth it. J Bone Joint Surg. 2018; 100: 556–63. 29613924 10.2106/JBJS.17.00621

[32] Bulman W , Dormans JP , Ecker ML , Drummond DS . Posterior spinal fusion for scoliosis in patients with cerebral palsy: A comparison of Luque rod and Unit Rod instrumentation. J Pediatr Orthop. 1996; 16: 314–23. 8728630 10.1097/00004694-199605000-00005

[33] Andersen GL , Hollung SJ , Klevberg GL , Kløve N . Norsk kvalitets‐ og oppfølgingsregister for cerebral parese (NorCP) – Årsrapport 2020 med plan for forbedringstiltak.

