# Supplementary figures and images for: Surgical and health outcomes of non‐ambulatory children with cerebral palsy and severe scoliosis: A population‐based, longitudinal study
Source: Dev Med Child Neurol. 2025 Aug 20;68(5):681–7. doi: 10.1111/dmcn.16473 (PMC13056022; doi:10.1111/dmcn.16473)

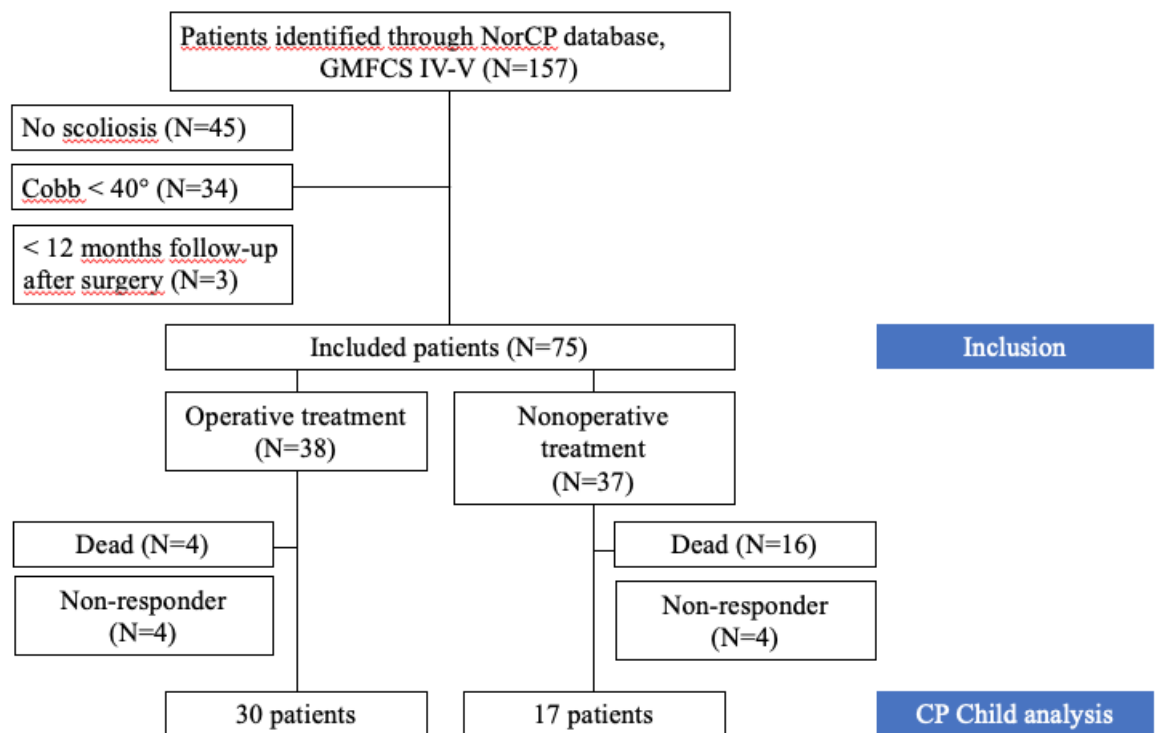

NorCP, Norwegian Quality and Surveillance Registry for Cerebral Palsy

Supplement: Supplementary file 1 — Figure S1: Inclusion and exclusion criteria for non‐ambulatory children with cerebral palsy and severe scoliosis. [file DMCN-68-681-s001.pdf]
